# Comparison of Active Electrode Materials for Non-Contact ECG Measurement

**DOI:** 10.3390/s19163585

**Published:** 2019-08-17

**Authors:** Shun Peng, Ke Xu, Wei Chen

**Affiliations:** Center for Intelligent Medical Electronics, School of Information Science and Technology, Fudan University, Shanghai 200433, China

**Keywords:** ECG monitoring, active electrode, flexible materials, signal quality indexes

## Abstract

For long-term and more convenience electrocardiograph (ECG) monitoring, an active- electrode-based ECG monitoring system, which can measure ECG through clothes, is proposed in this paper. The hardware of the system includes active electrodes, signal processing and data transmission modules and the software mainly includes a denoising algorithm based on empirical mode decomposition (EMD). Then the proposed system was verified using the comparison of the ECG signals measured synchronously by active electrodes and Ag/AgCl electrodes. In addition, three flexible materials, including conductive textile, copper foil tape and a flexible printed circuit (FPC) are developed for the sensing layer with active electrodes. To compare the performance of these three materials for the sensing layer, the ECG signals of 10 subjects were measured by different materials in three postures and several indexes for quality evaluation were calculated. Results show that effective and clear ECG waveforms can be measured by all three kinds of materials and the quality of ECG signals measured by FPC is the best by conducting a significant *t*-test for signal quality indexes of three materials.

## 1. Introduction

The ECG signal is one of the most commonly used signals in clinical examination. It can reflect normal cardiac electrical activity and diagnose heart-related diseases, such as atrial and ventricular hypertrophy, premature beats and atrial fibrillation 1Malik1996Heart. Passive electrodes, such as Ag/AgCl electrodes, are widely used in conventional ECG measurements. While passive electrodes can help obtain ECG signals with good quality, they have many limitations [1]. Firstly, skin preparation such as removing thick hair and the cuticle of the skin is required [2]. Secondly, wiping alcohol or applying conductive gel may cause skin irritation, especially in infants and sensitive populations [3,4]. Thirdly, conventional ECG measurements may cause panic and nervousness in patients, which indirectly affects the reliability of the measurement result [5]. Fourthly, conventional electrodes cannot be reused, thus resulting in a high measurement cost. The above limitations of conventional ECG measurement systems restrict their development towards long-term and daily monitoring [6].

Different from passive electrodes, active electrodes provide a means of ECG measurement through clothes which could be used in many applications and have therefore been further studied in recent years. Some attempts at ECG measurements in bathtubs and toilets, using an active electrodes were carried out but the signal quality was not good enough, so that only R peaks could be recognized [7,8]. The concept of a wearable device through the use of an active electrode was designed, and wireless communication was implemented using Bluetooth for easy data transfer and use [9,10]. However, these ECG measuring devices are not convenient for long-term wear because the electrodes are either fixed to the chest with a strap [9], or to two wrists [10]. Another attempt that uses the active electrode in ECG monitoring devices was carried out and a good quality ECG signal was achieved using offline denoising algorithms such as empirical wavelet transform [11] and singular spectrum analysis [12], whereas the online monitoring still remains a problem to be solved.

In the application of active electrodes, another concern is the comparison of signal qualities among different electrode materials for non-contact active ECG [13], and the compatibility between active electrodes and standard ECG measurement devices is seldom studied. In this paper, an ECG monitoring system based on an active electrode is proposed. It can measure an ECG signal through clothes on a chair, a recliner or a bed, which facilitates easy use and long-term ECG monitoring. The electronic components and signal output connector of the electrodes are carefully selected for easy compatibility with standard ECG measurement systems. In addition, the active electrode sensing layer in the system proposed is available in three flexible materials, including conductive textile, copper foil tape, and FPC.To compare the performance of these three materials for the electrode sensing layer, the ECG signals of 10 subjects were measured by active electrode made of different materials in three postures, including sitting, lying supine and side lying and several indexes for measure its quality were calculated.

This paper is organized as follows: Section 2 describes the principle of an active electrode and three flexible materials. Section 3 describes the system implementation including hardware design and software design and system verification. Section 4 illustrates the experimental setup for comparison among different electrode materials and the comparison result. Section 5 discusses this work and Section 6 gives the conclusion.

## 2. Materials and Methods

### 2.1. Active Electrode

**Figure 1:**
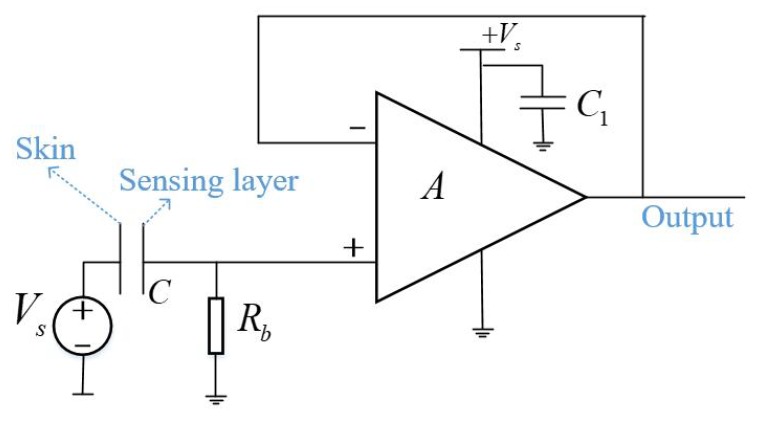
Equivalent electronic circuit of the active electrode.

Since the electrode and the skin are separated by a layer of clothes when measuring an ECG signal through clothes, the skin-sensing layer impedance is very large and for the passive electrode, the ECG signal is too susceptible to interference. To increase the anti-interference ability and load capacity of the electrode, a voltage follower is designed as a buffer. An equivalent electronic circuit of the active electrode is shown in Figure 1, where *A* is an operational amplifier used as a voltage follower with a gain of 1 and *Rb* is a bias resistor for the bias current of the operational amplifier. For electrical compatibility with standard ECG measurement systems, AD8605 (Analog Device Inc., Norwood, MA, USA) is selected as the operational amplifier because of its single-supply operation mode and low operating voltage.

The electrode sensing layer shown in Figure 1 is coupled with human skin into a capacitor *C* for sensing the electric potential changes of the skin surface. When measuring ECG, the electrode sensing layer is pressed by the back of the human body, so it needs to be made of flexible materials. In this paper, three flexible materials are available—and will be described in detail later—which are conductive textile, copper foil tape and FPC.

### 2.2. Three Materials

**Figure 2:**
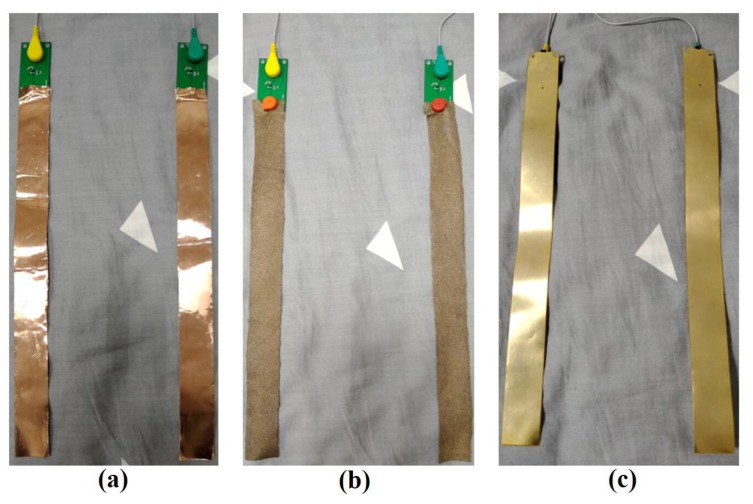
Three pairs of electrodes made of different materials: (**a**) copper foil tape, (**b**) conductive textile, (**c**) FPC.

The active electrode sensing layer is available in three flexible materials, including conductive textile, copper foil tape and FPC. Figure 2 shows three pairs of electrodes made of different materials.

The conductive textile tape used in this paper was purchased from Qingdao Tianyin Textile Technology Co., Ltd. (Qingdao, China). The basic material of the conductive textile is nylon spandex, which is implanted with silver ions by vacuum sputtering technology and the silver content is about 15%. Copper foil tape was purchased from Suzhou Aifeimin Shielding Conductive Material Co., Ltd. (Suzhou, China) and is made of conductive pressure-sensitive glue and purple copper. The FPC used in this paper was purchased from Shenzhen Hengxin Hengye Technology Co., Ltd. (Shenzhen, China). It is a kind of printed circuit made of flexible insulating substrate and its main materials are substrate, transparent glue and copper foil. The characteristics of these three materials are shown in Table 1.

## 3. System Implementation

### 3.1. Hardware Design

**Figure 3:**
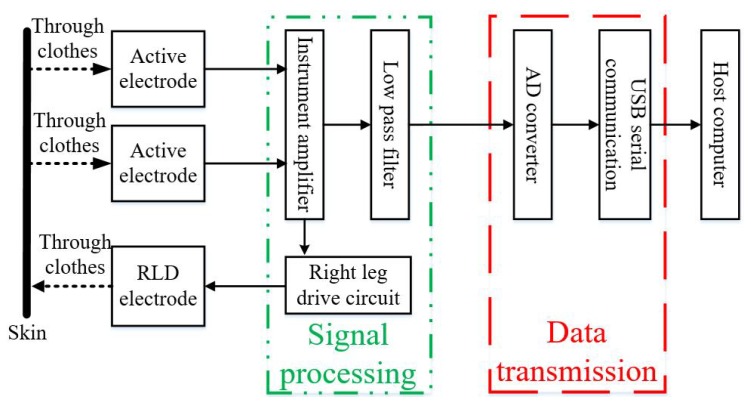
Hardware system block diagram.

The hardware of the system proposed consists of a sensor module, a signal processing module and a data transmission module, as shown in Figure 3.

The sensing module includes two active electrodes, as previously described, and a right leg drive (RLD) electrode. Two active electrodes are pressed on left and right sides of the back of human body through clothes to sense the electric potential changes of the skin surface. The RLD electrode is used to suppress the common mode signal of the two active electrodes and to reduce baseline wander. The RLD electrode is made by a piece of conductive textile and is pressed under the buttock through the clothes. In addition, the signal output connectors of the electrodes are designed as buttons to match the connector of the ECG measurement product.

The signal processing module includes an instrument amplifier, a right leg drive circuit and a low pass filter. An instrument amplifier is used to differentiate and amplify the output signals of the two active electrodes. The right leg drive circuit extracts common mode signals from the output signals of the two active electrodes and feeds them back to the human body to improve the common mode rejection ratio [14,15]. The data transmission module includes an AD conversion module and a 232 serial communication module. It is used to convert analog signals into digital signals and transmit them to the host computer.

### 3.2. Software Design

The host software functions include a parameter setting, signal processing, waveform display, data recording and so on. The signal processing part includes high pass digital filter and an EMD algorithm used to reduce noise [16,17,18], whose procedures are briefly described as follows.

Firstly, the ECG signals are filtered by a high pass digital filter with a 0.5 Hz cut-off frequency. Secondly, the filtered signal is decomposed by EMD and $n_{f}$ intrinsic mode functions (IMF) $c_{i}$ and one residual re are obtained. Then, the instantaneous frequency of each IFMare obtained by the Hilbert transform of each IFM and $n_{f}$ average frequencies $\bar{f}$ are calculated. Fourthly, dividing $n_{f}$ IFMs into two parts with a 30 Hz threshold: $k_{f}$ IMFs with high average frequency $\bar{f}$ and other IMFs with low average frequency $\bar{f}$. Finally, the output signal is calculated by the following formula:

(1) $$x_{out}=\sum_{i=k_{f}+1}^{n_{f}}c_{i}+re$$

**Figure 4:**
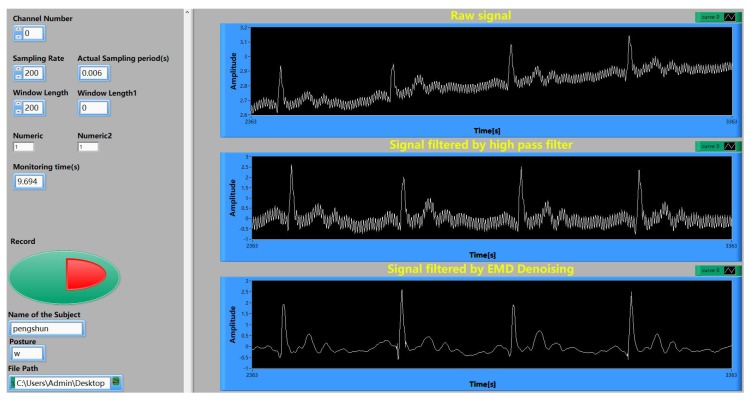
User interface of host computer software.

The host computer software is coded by Labview [19,20], and its user interface is shown in Figure 4.

### 3.3. System Verification

To verify the system proposed, an experiment designed to compare the signals measured by the system proposed and conventional electrode was carried out. In this experiment, the sensing layer of active electrode is made of FPC. In addition, an Ag/AgCl electrode was selected as the gold standard of comparison and the ECG signal measured by it was used as the reference signal.

The experimental procedures involving human subjects described in this paper were approved by the Institutional Review Board.

**Figure 5:**
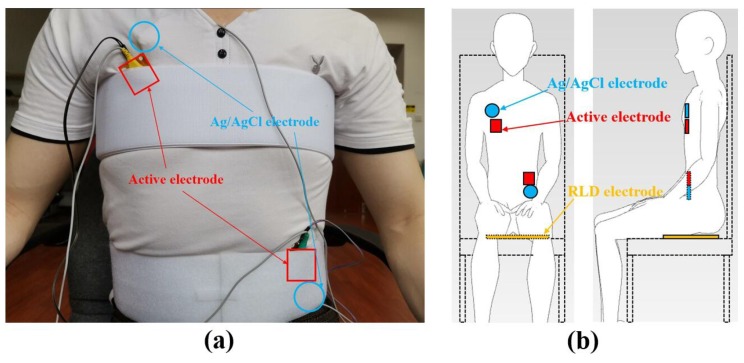
Experimental setup of system verification experiment: (**a**) picture, (**b**) schematic.

To be able to measure the ECG signal of the standard lead, the active electrodes are made into shapes similar in size to those of the Ag/AgCl electrode, and are placed on the front of the body of the subject with straps. The electrode setup adopts the standard II lead mode, that is, the positive electrode (the electrode connected to the positive end of the differential amplifier) is placed on the right chest and the negative electrode is placed on the left waist. The Ag/AgCl electrodes are attached to the skin and the active electrodes are fixed on the outside of the clothes with straps. During the experiment, the subject sits in a chair and the RLD electrode made of conductive cloth is pressed by the buttocks to suppress the common mode signal of the two electrodes and improve the quality of the ECG. The experimental setup is shown in Figure 5.

To compare the signals measured by the active electrode and the Ag/AgCl electrode, two kinds of indexes, the time domain waveform correlation and the characteristic parameter accuracy are introduced in this paper. The time domain waveform correlation index is expressed by the Pearson correlation coefficient *r* [21,22,23], which is defined by

(2) $$r=\frac{n\sum x_{i}y_{i}-\sum x_{i}\sum y_{i}}{\sqrt{n\sum x_{i}^{2}-{\left(\sum x_{i}\right)}^{2}}\sqrt{n\sum y_{i}^{2}-{\left(\sum y_{i}\right)}^{2}}}$$

where *x* and *y* are two ECG sequences respectively. Correlation coefficients *r* in the range of 0.8 to 1 indicate a strong correlation between the two segments of the ECG waveform.

In addition, the instantaneous heart rate, which is a physiological parameter, is extracted from the ECG sequence (in this paper, the instantaneous heart rate of ECG signal is calculated by R-R interval). Assuming that an ECG sequence contains *m* ECG cycles, then the instantaneous heart rate is a sequence of length. In this paper, the Pearson correlation coefficient *r* and the root mean square error (*RMSE*) of the instantaneous heart rate corresponding to two segments of the ECG signal are calculated respectively to express the accuracy of the characteristic parameters of the two ECG signals [24]. The mean square error is defined by

(3) $$RMSE=\sqrt{\frac{1}{m}{\left(x_{i}-y_{i}\right)}^{2}}$$

**Figure 6:**
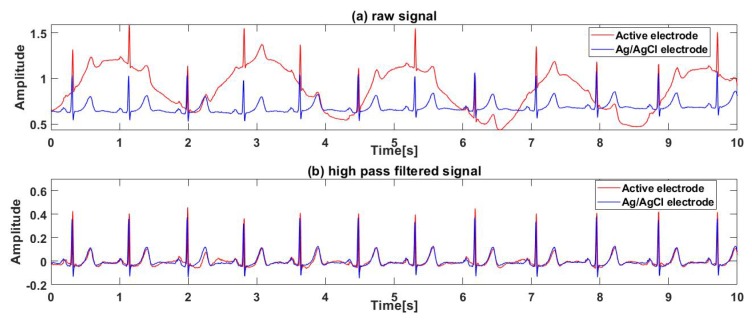
Comparison between signals measured by active electrodes and Ag/AgCl electrodes: (**a**) raw signal, (**b**) high pass filtered signal.

Figure 6 shows one subject’s ECG signal measured by an active electrode and an Ag/AgCl electrode synchronously. Figure 6a shows the raw ECG signals measured by two kinds of electrodes. Since the entire system proposed is powered by a lithium battery, the high frequency noise in the raw signal is very small but there is a certain baseline wander in the raw signal, especially in a signal measured by the active electrode. It is obvious that the baseline wander is caused by breathing, according to the raw signal waveform. Figure 6b shows the signal filtered by a high pass filter. It can be seen that the ECG signal measured by active electrodes through clothes contains clear P, QRS, T detail waveforms, and is very similar to the reference ECG signal measured by Ag/AgCl electrodes synchronously.

**Figure 7:**
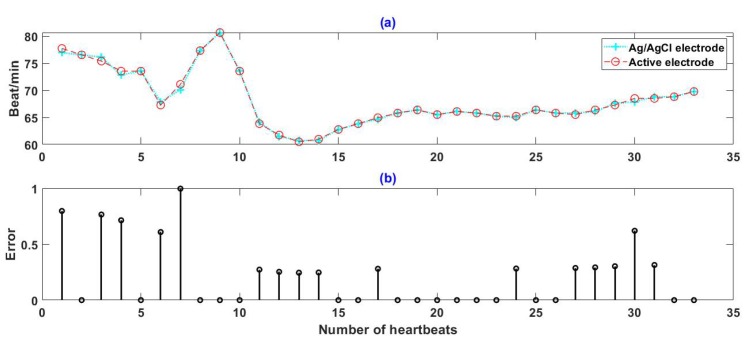
Comparison between instantaneous heart rate obtained by active electrode and Ag/AgCl electrode: (**a**) instantaneous heart rate, (**b**) the error between instantaneous heart rate obtained by two kinds of electrode.

The ECG sequence with a length of 30 s was intercepted from the experimental results and the instantaneous heart rate was calculated with R-R interval. Results are shown in Figure 7, where Figure 7a shows the comparison of the instantaneous heart rate between signals measured by two kinds of electrode and the Figure 7b shows the error between them. It can be seen that the instantaneous heart rate obtained by active electrodes is very similar to that of Ag/AgCl electrodes. Within the 35 heartbeats shown in the Figure 7, the absolute error of instantaneous heart rate does not exceed 1 beat/min.

To quantify the similarity of the waveforms obtained by the active electrode and the Ag/AgCl electrode, the ECG sequence with a length of 30 s was intercepted from the ECG signals with baseline wander eliminated and the indexes given in Equation (3) were calculated. The calculation results are shown in Table 2, where $r_{w}$ represents the correlation coefficient of waveform between signals measured by the active electrode and the Ag/AgCl electrode $r_{HR}$ represents the correlation coefficient of instantaneous heart rate obtained by two kinds of electrodes and $RMSE_{HR}$ represents the mean square error of the instantaneous heart rate.

It can be seen from Table 2 that the correlation coefficients of the waveforms $r_{w}$ are above 0.8, which indicates that the ECG of the active electrode is strongly correlated with that of the Ag/AgCl electrode. In addition, the instantaneous heart rate correlation coefficients are also above 0.98 and the mean square errors are lower than 1 beat/min.

**Figure 8:**
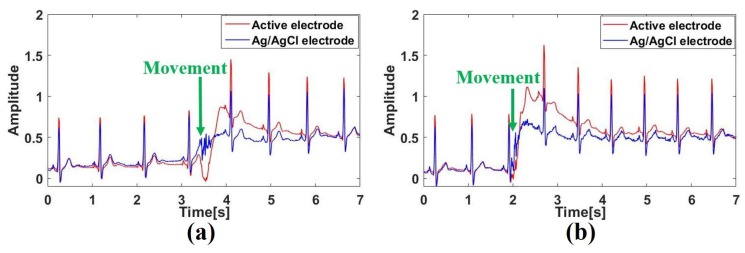
ECG measurement under turning movement of upper body: (**a**) left turning movement of upper body, (**b**) right turning movement of upper body.

Studies have shown that biomedical signals like ECG are susceptible to motion interference [25]. For this reason, an experiment in which the active electrode measures the ECG signal under body movement was carried out while being compared with the Ag/AgCl electrode. In the experiment, to avoid respiratory effects, the subject sat in the chair and held his breath for 15 s. The position of the active and conventional electrodes remained the same as previously mentioned, but in the ECG measurement, the subject was required to complete a sudden turning movement of upper body. Figure 8 shows the ECG measurement results of a subject under two movements.

As can be seen from Figure 8, the turning movements affect both the signal of the active electrode and the signal of the Ag/AgCl electrode and causes the jump of the signal value. Among them, the change of the active electrode ECG signal is more intense and the time to restore stability after the change is also longer. Therefore, relatively speaking, the active electrode is more seriously affected by the movement because the signal of the active electrode is measured through the clothes. Generally speaking, the influence of the signal is not great and the signal can be recovered quickly.

**Figure 9:**
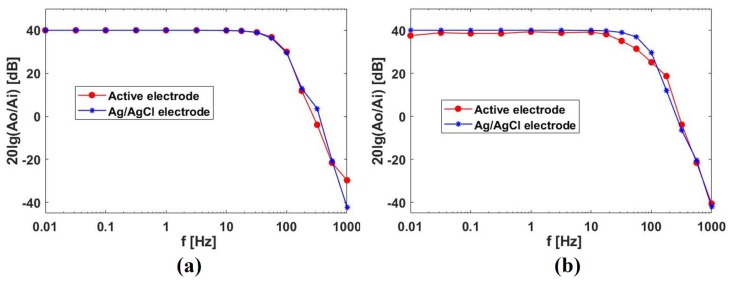
Amplitude-frequency response of active electrode system and passive electrode system: (**a**) electrodes of the active electrode system measure signals directly, (**b**) electrodes of the active electrode system measure signals through clothes(a simple cotton underwear about 1 mm in thickness).

**Figure 10:**
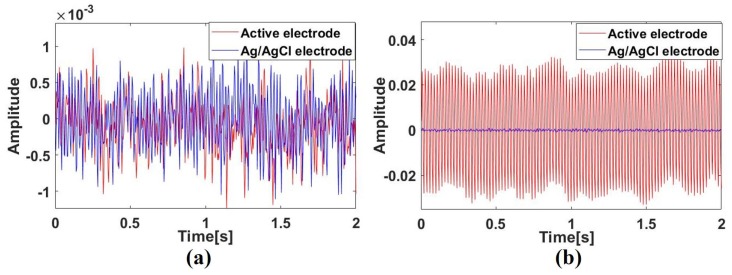
Output noise of system under short connection of electrodes: (**a**) electrodes of the active electrode system short connect directly, (**b**) electrodes of the active electrode system short connect through clothes (a simple cotton underwear about 1 mm in thickness).

The results in Figure 8 imply that the bandwidth of a system with active electrodes is smaller than that of an Ag/AgCl electrodes system. To compare the bandwidth of the two systems more clearly, bandwidth response diagrams are given [26], as shown in Figure 9. The data in this figure were measured and processed by inputting a set of sinusoidal signals of specified frequencies into the system. In addition, the output noise of the system under the condition of short connection of positive and negative electrodes is tested, as shown in Figure 10.

It can be seen from Figure 9 and Figure 10 that when electrodes of the active electrode system measure a signal or short connect directly, the amplitude-frequency response of the system is similar to that of the Ag/AgCl electrode system and the noise amplitude of the output is also similar. However, when electrodes of the active electrode system measure signal or short connect through clothes, the amplitude-frequency response curve of the system is slightly lower than the Ag/AgCl electrode system at low frequencies and the output noise amplitude is larger than that of the Ag/AgCl electrode system. This shows that the signal measured by electrodes through clothes will cause the signal amplitude to decrease and the noise to increase.

In summary, the performance of the active electrode system is slightly worse than that of the Ag/AgCl electrode system when active electrodes measure the signal through clothes, but the difference is not large. From the actual ECG signal measured by the system proposed in Figure 6, it can be inferred that under the experimental setup described in this section, the ECG signal measured by the active electrode through clothes, though affected by breathing, still contains the basic features and most of the waveform details. Also, the physiological parameters extracted from active-electrode-based ECG waveform have high accuracy, which can meet the requirements of cardiac examination and disease diagnosis.

## 4. Experiments and Results

In the ECG measurement experiment of Section 3, the fixing method of active electrodes is not convenient for daily use, because they need to be placed on the front of the human body with a strap. To be convenient for daily ECG measurements, the active electrodes could be placed on a chair, a recliner or a bed. In detail, two active electrodes are pressed under two sides of the back of the subject’s body and sense the potential change of the back skin through the clothes. The RLD electrode, made of a conductive textile, is pressed under the buttock, and feeds back the common mode signal of two electrodes to subject’s body through the clothes to improve the common mode rejection ratio (CMMR). In this way, the system proposed in this paper can be used for ECG monitoring of driving, sleeping, daily office, and so forth.

**Figure 11:**
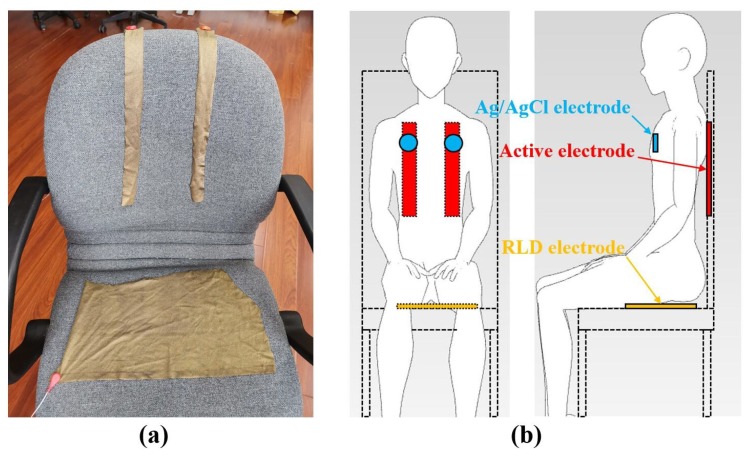
Experimental setups in sitting posture: (**a**) picture, (**b**) schematic.

**Figure 12:**
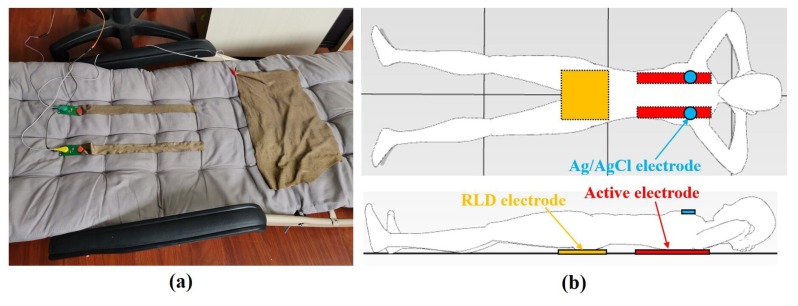
Experimental setups in lying supine posture: (**a**) picture, (**b**) schematic.

**Figure 13:**
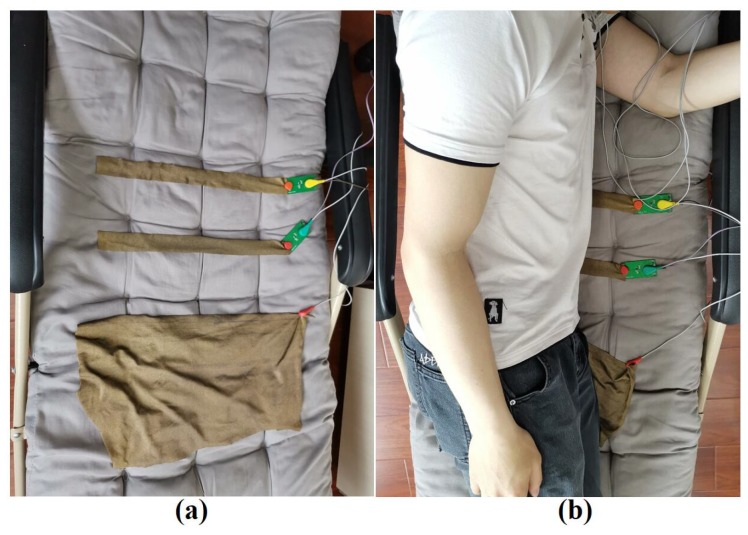
Experimental setups in side lying posture: (**a**) before the subject side lied on the recliner, (**b**) the subject was side lying on the recliner.

To compare the performances of the three materials, experiments which measure the ECG signal with different kinds of electrodes made of the three materials were carried out. In the experiment, the subjects were seated on a chair and laid on a recliner, as shown in Figure 11 and Figure 12. Ag/AgCl electrodes were also used to measure ECG signal synchronously and to function as the reference signal. Since the active electrode lead is similar to the standard I lead, the Ag/AgCl electrode is also placed according the standard I lead, which means that the two Ag/AgCl electrodes are attached to the left and right chest, respectively. In addition, side lying is a very common but not easy to measure ECG posture during sleep, so it has also been added to the experimental setup in this section to illustrate the superiority of the system proposed, as shown in Figure 13.

To increase the reliability of the experiment, there were 10 subjects in the experiment, including 6 males and 4 females.The basic information of the subjects is listed in Table 3.

### 4.1. Sitting

**Figure 14:**
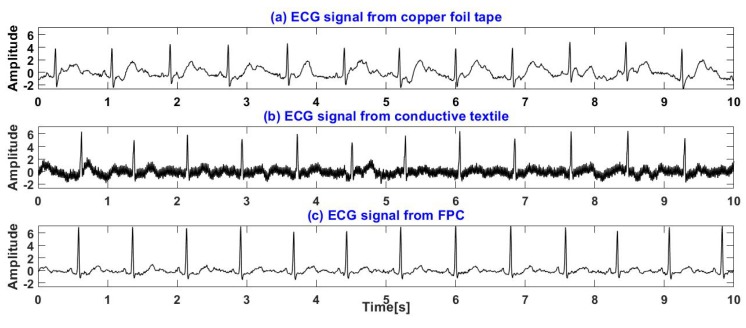
ECG signals of a subject sitting on a chair: (**a**) ECG signals measured by copper foil tape. (**b**) ECG signals measured by conductive textile. (**c**) ECG signals measured by FPC.

Figure 11 shows the experimental setup of the ECG measurement of the subject sitting on a chair, where two active electrodes are placed on the backrest of the chair and the RLD electrode, made of a conductive textile, is placed on the chair. In addition, two Ag/AgCl electrodes were attached to the human skin to measure ECG signals using standard I lead. Figure 14 shows the ECG signals of a subject sitting in a chair measured through the clothes by electrodes made of three kinds of materials proposed, where Figure 14a–c shows the ECG signals measured by copper foil tape, conductive textile and FPC, respectively. It can be seen that, in this experiment, the ECG signal measured by the electrode, made of conductive textile, contains more high frequency noise. Although the signal measured by the electrode made of copper foil tape has little high frequency noise, the detailed waveform such as P wave is not very clear. There are other spikes near the T wave, which will cause false detection of T wave, and the R wave amplitude is also small. The signals measured by the electrodes made of FPC contain clear QRS, T and P waves and are unaffected by high frequency noise.

Studies have shown that kurtosis can be used to measure the quality of ECG signals [27,28]. To compare the quality of signals measured by three materials, kurtosis was added as an index to measure the quality of ECG signals. The kurtosis of clean ECG signals is greater than 5 [29], and the kurtosis of ECG signals contaminated by baseline wander or high frequency noise may be less than 5 [30]. The kurtosis *k*, of signal *x*, is defined as follows:

(4) $$k=\frac{1}{n}\sum_{i=1}^{n}{\left[\frac{x_{i}-\mu_{x}}{\sigma }\right]}^{4}$$

where σ is the standard deviation of *x*, $\mu_{x}$ is an empirical estimate of *x*. The lower the kurtosis, the worse the quality of the ECG signal.

In addition, the signal-to-noise ratio (*SNR*) is introduced as an index to measure the signal quality of active electrodes [31], which is defined as:

(5) $$SNR=10lg(\frac{\sum_{i=1}^{n}{\left(y_{i}\right)}^{2}}{\sum_{i=1}^{n}{\left(x_{i}-y_{i}\right)}^{2}})$$

where $y_{i}$ is the reference ECG signal measured by the Ag/AgCl electrodes. Since the Ag/AgCl electrode is attached to the chest of the subject, which is not the same as the position of the active electrode, the SNR calculated by Equation (5) is not a true signal-to-noise ratio, and is smaller than that, but it can also reflect the signal’s quality.

The ECG sequence with a length of 30 s was intercepted from the ECG signals of 10 subjects, then their kurtosis and SNR were calculated as shown in Table 4. The correlation coefficients $r_{HR}$ of the instantaneous heart rate obtained by active electrodes and Ag/AgCl electrodes are also shown in Table 4.

It can be seen from Table 4 that $r_{HR}$ of ECG signals measured by electrodes made of three materials are close to 1, which indicates that the instantaneous heart rate obtained by the electrodes made of three materials is very accurate. However, the kurtosis and SNR of the ECG signals measured by the three materials are quite different. The blue numbers in Table 4 show that the kurtosis of the ECG signal measured by the copper foil tape on six subjects is the smallest, while the red numbers show that the kurtosis of the ECG signal measured by the FPC on seven subjects is the largest. To verify whether there is a difference in the kurtosis and SNR of the signals measured by the three materials, the *t*-test is used in this paper and the calculation results are shown in Table 5.

It can be seen from Table 4 that the mean kurtosis value of the signal measured by the FPC electrode is larger than those of the other two materials and the SNR mean value of the signal measured by the FPC electrode is larger than that of the copper foil tape. Therefore, it can be inferred that the signal quality measured by the FPC electrode is better than those of the other two electrode materials when sitting on a chair.

### 4.2. Lying Supine

Figure 12 shows the experimental setup of the ECG measurement, in which the subject lies supine on a recliner, where two active electrodes are pressed under two sides of the back of the subject’s body and the RLD electrodes made of a conductive textile are pressed under the buttock. In addition, two Ag/AgCl electrodes were attached to the human skin to measure ECG signals using standard I lead.

**Figure 15:**
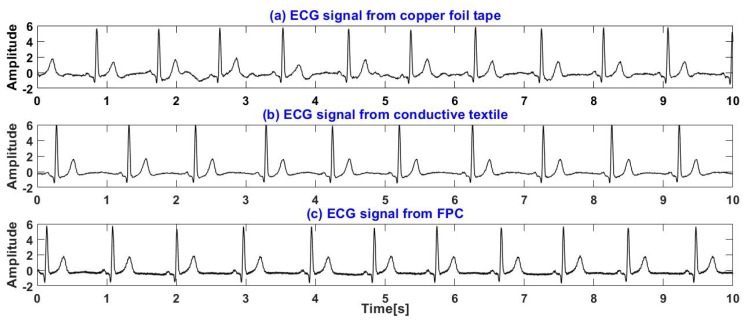
ECG signals of a subject lying supine on a recliner: (**a**) ECG signals measured by copper foil tape. (**b**) ECG signals measured by conductive textile. (**c**) ECG signals measured by FPC.

Figure 15 shows the ECG signals of a subject lying supine on a recliner measured through clothes by electrodes made of the three kinds of materials proposed, where Figure 15a–c shows the ECG signals measured by copper foil tape, conductive textile and FPC, respectively.

As can be seen from Figure 15, unlike with sitting on the chair, since the electrode is pressed tighter when lying supine, the ECG measured by the electrode made of the conductive cloth no longer has a lot of high frequency noise. In addition, clear T waves can be detected in the ECG signals of the three materials. Therefore, the T-T interval is extracted from the ECG signal and its correlation coefficient $r_{TT}$ with the reference signal is calculated as an index to measure the quality of the signal. The ECG sequence with a length of 30 s was intercepted from the ECG signals of 10 subjects, then their kurtosis, SNR, $r_{HR}$ and $r_{TT}$ were calculated as shown in the Table 6.

It can be seen from Table 6 that the instantaneous heart rate correlation coefficient $r_{HR}$ of ECG signals measured in the lying supine posture with three materials is close to 1, which shows that the instantaneous heart rate obtained in the lying supine posture with three materials is very accurate. However, the kurtosis, SNR and $r_{TT}$ of the ECG signals measured by the three materials are quite different. For example, the blue numbers in Table 6 indicate that the SNR of the ECG signal measured by the copper foil tape on the 8 subjects is the smallest and the red numbers indicate that the SNR of the ECG signal measured by the FPC on the 8 subjects is the largest. To verify whether there is a difference in the kurtosis, SNR and $r_{TT}$ of the signals measured by the three materials, the *t*-test is used in this paper and the calculation results are shown in the Table 7.

As can be seen from Table 7, the kurtosis of ECG signal measured by conductive textile is larger than that of the copper foil tape, and the SNR of ECG signal measured by FPC is larger than that of the copper foil tape. In addition, the $r_{TT}$ of the signal measured by FPC is larger than that of the other materials. This result shows that the T wave of the ECG signal measured by the FPC electrode is most accurate when lying supine, so FPC is recommended as the electrode material to measure ECG through clothes when lying supine.

### 4.3. Side Lying

Figure 13 shows the experimental setup of the ECG measurement of the subject lying on their side on a recliner, where two active electrodes were placed on the waist of the subject lying on their side, and the RLD electrodes made of a conductive textile are pressed under buttock. In addition, two Ag/AgCl electrodes were attached to the human skin to measure ECG signals using standard I lead.

**Figure 16:**
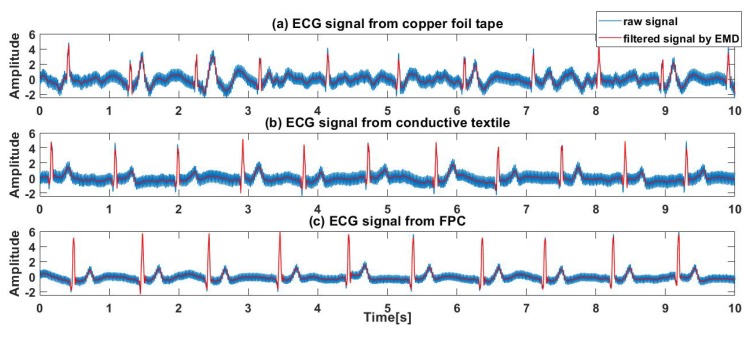
ECG signals of a subject side lying on a recliner: (**a**) ECG signals measured by copper foil tape. (**b**) ECG signals measured by conductive textile. (**c**) ECG signals measured by FPC.

Figure 16 shows the ECG signals of a subject lying on their side on a recliner measured through the clothes by electrodes made of the three kinds of materials proposed, where Figure 16a–c shows the ECG signals measured by copper foil tape, conductive textile and FPC, respectively.

It can be seen from Figure 16 that the ECG signal measured by the active electrode through the clothes when subjects are lying on their side contains more high frequency noise but after it is filtered by EMD denoising, clear QRS waves can be obtained. The signal of the conductive textile and the FPC is better and clear T waves can be obtained but there is a small amount of spike interference near the T wave in the signal of the copper foil tape.

The ECG sequence with a length of 30 s was intercepted from the ECG signals of 10 subjects, then their kurtosis, SNR, $r_{HR}$ were calculated as shown in Table 8.

It can be seen from Table 8 that the instantaneous heart rate correlation coefficient $r_{HR}$ of ECG signals measured when subjects lie on their side on the recliner is close to 1, which shows that the instantaneous heart rate obtained in the side lying posture is very accurate. In addition, the SNRs of the ECG signals measured by the three materials are very small, because the position of the active electrodes is very different from that of the Ag/AgCl electrodes when the subject is lying on their side on the recliner, so the signals of the two electrodes are not comparable in waveform. However, the kurtosis of the ECG signals measured by the three materials is quite different. As can be seen in Table 8, the blue numbers represent that the kurtosis of the ECG signal measured by the copper foil tape on the 7 subjects is the smallest. To verify whether there is a difference in the kurtosis of the signals measured by the three materials, the *t*-test is used, and the calculation results are shown in Table 9.

As can be seen from Table 9, the kurtosis of the ECG signal measured by copper foil tape is smaller than that of the other materials. Therefore, it can be inferred that the signal quality measured by the copper foil tape electrode is worse than that of the other two electrode materials when lying on the side on the recliner.

## 5. Discussion

The system proposed is mainly used to measure the ECG signal on the back of the human body through the clothes. From the waveforms of the ECG signals measured in the three postures of sitting, lying supine and side lying in Section 4, the system proposed in this paper can accurately detect the R wave. As we know, the R-R interval is a very important characteristic parameter of the ECG signal for deriving many physiological indexes such as heart rate variability [32]. The correlation coefficient $r_{HR}$ between the instantaneous heart rate calculated by the R-R interval measured by the system proposed and the reference ECG signal is very high (close to 1). This proves that the R-R interval extracted from the ECG signal measured by this system proposed is very accurate. What is more, the system with the FPC material can get obvious P, QRS and T waves in both sitting and lying postures, so it can meet the requirements of the diagnosis of arrhythmia and the prediction of heart disease.

In addition, the SNR calculated in Section 4 can only be used as an index to compare the quality of ECG signals measured by the three materials, rather than the true SNR. This is because the ECG waveform measured by the active electrode is different from that measured by the Ag/AgCl electrode under lead I, as shown in Table 10.

Table 10 shows the correlation coefficient $r_{w}$ between ECG waveform measured by active electrode and reference signal waveform from experimental data of subject 1-4 in lies supine posture, and it can be seen that most of them are small. In Section 3, a high correlation coefficients $r_{w}$ was obtained when the active electrodes were placed on the front of the body of the subject with straps, so the fact that the correlation coefficient is low here is due to the great difference in the position of the active electrodes and the Ag/AgCl electrodes on the human body.

Section 4 compares the quality of ECG signals measured by three active electrode materials in three postures. The conclusions are as follows: the quality of the ECG signal measured by the FPC electrode material is better than that of the other two electrode materials when sitting, and the T wave of the ECG signal measured by the FPC electrode material is the most accurate when lying supine, and the quality of the ECG signal measured by the FPC material and the conductive cloth material is better than that of the copper foil tape. Therefore, FPC enables the best ECG signal quality, followed by conductive cloth material, and the worst material is the copper foil tape because it has the lowest signal quality in every posture.

Considering the measurement performance, FPC is the best choice for the active electrode sensing layer. However, the conductive textile is the only material that is stretchable and can be kneaded of the three materials, thus it is also a good choice for the active electrode sensing layer.

From the ECG waveforms of the measurements in three postures, it can be inferred that the quality of the ECG signal is best when lying supine, with clear QRS, T and P waves obtained from the ECG signal. This may result from the fact that the active electrode is pressed the tightest onto the back of the human body when lying supine. The quality of ECG signals while sitting in a chair is also acceptable, where clear QRS and T waves can be obtained, but P waves are sometimes not obvious. The quality of the ECG signal is the worst when lying on the side and the calculated SNR is also the smallest. Due to the influence of the position on the electrodes, the P wave was almost invisible in the ECG signal measured by 10 subjects when side lying and the T wave was also not detected in some subjects’ ECG signals.

The system in this paper has been able to measure the ECG signal through very thick clothes. But there are still many problems to be solved in order to achieve clinical application. Here are a few questions the author thought of:

Firstly, if ECG signals are monitored during sleep, how can ECG signals still be measured when patients move their bodies, such as turning over? The author thinks that bigger electrode can be designed, or more signal channels can be added to the system by using more electrodes.

Secondly, the system measures the ECG signal by placing electrodes on the back of the body, resulting in a difference between the measured signal and that of the standard lead.

Thirdly, how to prevent the electrode from getting dirty? Or how to clean after the electrode is dirty? One way is to put a washable textile on the electrode to prevent it from getting dirty. If the electrode is dirty, the electrode made of the FPC can be wiped with alcohol, and the electrode made of conductive textile can be washed with water.

Finally, under certain postures, the ECG signal is seriously contaminated by noise, thus it is necessary to filter the signal. However, the filtered signal by the proposed system has a certain delay. To display a clearer ECG signal in real time when lying on the side, it is necessary to further improve the denoising algorithm in terms of real-time performance.

## 6. Conclusions

This paper mainly introduces a system for ECG measurement through clothes, and further proposes some comparison among different active electrode materials. Firstly, the whole design of the ECG measurement system is introduced, including hardware design and software design. To verify the system proposed, active electrodes were fixed onto the front of the body of the subject with straps to measure the ECG signal, with the reference signal measured by using the Ag/AgCl electrode synchronously. The results show that the waveform and instantaneous heart rate of the ECG signal measured by the active electrode are very similar to those of the Ag/AgCl electrode. Then, three kinds of materials which are available for the active electrode sensing layer, including conductive cloth, copper foil tape and FPC, are introduced, and ECG signals were measured with an active electrode made of different materials in three postures, including sitting, lying supine and lying on the side. To compare the performance of different materials for the electrode sensing layer, the quality of the ECG signals was measured by two indexes including kurtosis and SNR. The results show that clear ECG waveforms can be measured by all three kinds of electrode materials, but the quality of the ECG signals measured by FPC is significantly better.

## Figures and Tables

**Table 1:** Characteristics of three materials.

Material Name	Conductive Textile	Copper Foil Tape	FPC
Base material	Nylon spandex	Purple copper	Polyimide
Material component	Silver	Acrylic glue	Copper
Thickness	0.5 mm	0.1 mm	0.15 mm
Resistivity	2 Ω·cm	0.15 Ω·cm	0.08 Ω·cm
Stretchability	Stretchable (all sides)	Non-stretchable	Non-stretchable
Bendability	Bendable	Bendable	Bendable
Cleanability	Washable	Can be wiped with alcohol	Can be wiped with alcohol

**Table 2:** Signal indexes for comparison between the active electrode and the Ag/AgCl electrode.

Subject	$r_{w}$	$r_{\mathrm{HR}}$	${\mathrm{RMSE}}_{\mathrm{HR}}$ (Beat/min)
1	0.822	0.994	0.229
2	0.804	0.966	0.406
3	0.877	0.986	0.394
4	0.872	0.998	0.361

**Table 3:** Basic informations of subjects.

Subject Number	1	2	3	4	5	6	7	8	9	10
Gender	male	male	male	female	female	male	female	male	male	female
Age	34	23	26	23	20	23	25	26	24	21
Weight	75	69	61	55	50	70	51	60	65	57

**Table 4:** Quality indexes of ECG signals measured on a chair. (The blue number show that the kurtosis of the copper foil tape on six subjects is the smallest, and the red numbers show that the kurtosis of the FPC on seven subjects is the largest.)

Subject Number		1	2	3	4	5	6	7	8	9	10
kurtosis	tape	22.56	**12.19**	19.10	**6.12**	**5.52**	26.70	**5.83**	**14.27**	10.95	**5.55**
	textile	25.69	13.13	18.17	12.74	13.22	24.93	8.36	15.58	6.15	7.23
	FPC	23.31	**25.03**	19.57	**13.63**	**26.88**	22.78	**12.07**	**20.99**	**17.19**	**11.67**
SNR	tape	0.04	0.41	2.33	−0.77	−1.53	0.18	−0.25	1.90	−2.22	−1.29
	textile	0.13	0.65	2.70	1.29	−0.64	0.36	1.41	1.63	−0.93	1.24
	FPC	−0.31	1.24	2.68	−0.45	0.14	0.30	1.61	2.56	0.79	2.55
$r_{HR}$	tape	0.996	0.993	0.993	0.996	0.991	0.997	0.992	0.998	0.998	0.976
	textile	0.995	0.973	0.997	0.998	0.985	0.995	0.991	0.998	0.995	0.987
	FPC	0.996	0.998	0.996	0.993	0.993	0.999	0.980	0.997	0.998	0.988

**Table 5:** Significant *t*-test of signal quality indexes of three materials (alpha = 0.05).

Indexes	Hypothesis	*p*	h
kurtosis	$k_{FPC}>k_{tape}$	0.0224	1
	$k_{FPC}>k_{textile}$	0.0496	1
	$k_{textile}>k_{tape}$	0.3097	0
SNR	$SNR_{FPC}>SNR_{tape}$	0.0264	1
	$SNR_{FPC}>SNR_{textile}$	0.267	0
	$SNR_{textile}>SNR_{tape}$	0.0659	0

**Table 6:** Quality indexes of ECG signals measured in the lying supine posture. (The blue number show that the SNR of the copper foil tape on eight subjects is the smallest, and the red numbers show that the SNR of the FPC on eight subjects is the largest.)

Subject Number		1	2	3	4	5	6	7	8	9	10
kurtosis	tape	25.40	10.28	16.63	6.84	4.59	9.95	11.94	17.81	3.80	4.44
	textile	26.54	19.67	20.46	20.98	22.32	15.69	7.09	19.39	16.69	3.00
	FPC	24.39	27.80	15.98	19.61	21.53	9.58	7.78	17.62	11.62	7.95
SNR	tape	0.07	**0.17**	**1.89**	**−0.57**	**−1.82**	**0.76**	**1.61**	3.36	**−1.39**	**−0.70**
	textile	0.05	2.04	2.42	1.40	0.63	0.79	2.64	3.21	0.94	−0.46
	FPC	**0.21**	1.83	**2.72**	**2.91**	**1.09**	**1.08**	**2.80**	**3.56**	−1.45	**2.20**
$r_{HR}$	tape	0.999	0.996	0.997	0.969	0.985	0.999	0.999	0.999	0.997	0.991
	textile	0.993	0.993	0.999	0.999	0.996	0.997	0.997	0.999	0.998	0.923
	FPC	0.999	0.999	0.997	0.998	0.997	0.999	0.997	0.999	0.994	0.982
$r_{TT}$	tape	0.419	0.308	0.960	0.578	0.511	0.976	0.953	0.990	0.482	0.605
	textile	0.584	0.983	0.987	0.872	0.145	0.972	0.980	0.991	0.100	0.898
	FPC	0.930	0.998	0.964	0.932	0.852	0.931	0.982	0.983	0.932	0.982

**Table 7:** Significant *t*-test of signal quality indexes of three materials (alpha = 0.05).

Indexes	Hypothesis	*p*	h
kurtosis	$k_{FPC}>k_{tape}$	0.0570	0
	$k_{FPC}>k_{textile}$	0.5979	0
	$k_{textile}>k_{tape}$	0.0364	1
SNR	$SNR_{FPC}>SNR_{tape}$	0.0331	1
	$SNR_{FPC}>SNR_{textile}$	0.2969	0
	$SNR_{textile}>SNR_{tape}$	0.0600	0
$r_{TT}$	$r_{FPC}>r_{tape}$	0.0025	1
	$r_{FPC}>r_{textile}$	0.0482	1
	$r_{textile}>r_{tape}$	0.3035	0

**Table 8:** Quality indexes of ECG signals measured when subjects are lying on their side. (The blue number show that the kurtosis of the copper foil tape on seven subjects is the smallest.)

Subject Number		1	2	3	4	5	6	7	8	9	10
kurtosis	tape	**9.18**	**10.02**	**4.29**	**5.19**	4.320	13.02	**10.27**	**8.58**	7.29	**3.55**
	textile	25.87	21.03	7.27	19.90	4.11	10.02	15.86	14.38	6.59	9.22
	FPC	26.98	24.71	14.28	5.84	7.453	11.51	16.72	13.54	8.92	8.38
SNR	tape	−0.87	−0.91	−1.37	−1.08	−2.87	−0.64	−0.13	0.38	−0.54	−0.23
	textile	−0.33	−0.14	0.18	−2.75	−2.74	−0.53	0.35	0.85	−0.13	−0.11
	FPC	−0.71	0.70	0.76	−1.94	−2.02	−0.55	0.14	0.91	−0.47	−0.15
$r_{HR}$	tape	0.998	1	0.988	0.538	0.986	0.998	0.997	1	0.984	0.538
	textile	0.993	1	0.984	0.388	0.975	0.998	0.998	0.996	0.997	0.477
	FPC	0.927	0.999	0.996	0.973	0.991	0.996	0.987	0.997	0.984	0.814

**Table 9:** Significant *t*-test of signal quality indexes of three materials (alpha = 0.05).

Indexes	Hypothesis	*p*	h
kurtosis	$k_{FPC}>k_{tape}$	0.0106	1
	$k_{FPC}>k_{textile}$	0.4503	0
	$k_{textile}>k_{tape}$	0.0147	1

**Table 10:** Correlation coefficient of waveform of ECG signals measured in the lying supine posture.

Subject Number		1	2	3	4
$r_{w}$	tape	0.475	0.465	0.827	0.411
	textile	0.421	0.797	0.852	0.767
	FPC	0.462	0.755	0.865	0.870

## Abbreviations

The following abbreviations are used in this manuscript:

ECG	Electrocardiograph
EMD	Empirical mode decomposition
FPC	Flexible printed circuit
RLD	Right leg drive
IMF	Intrinsic mode functions
HIT	Hilbert transform
RMSE	Root mean square error
CMRR	Common mode rejection ratio
SNR	Signal-to-noise ratio
